# Isolated leptomeningeal angiomatosis in the sixth decade of life, an adulthood variant of Sturge Weber Syndrome (Type III): role of advanced Magnetic Resonance Imaging and Digital Subtraction Angiography in diagnosis

**DOI:** 10.1186/s12883-020-01944-5

**Published:** 2020-10-06

**Authors:** Vetrivel Muralidharan, Gaetano Failla, Mario Travali, Tiziana Liliana Cavallaro, Marco Angelo Politi

**Affiliations:** 1 Fellow in Advanced Training in Neuroendovascular Interventions, Department of Medical Surgical Science and Advanced Technologies “GF Ingrassia”, University of Catania, Catania, Italy; 2 Department of Neurology, Cannizzaro Hospital, Catania, Italy; 3 Resident in Radiology, Department of Medical Surgical Science and Advanced Technologies “GF Ingrassia”, University of Catania, Catania, Italy; 4 Diagnostic & Interventional Neuroradiology, Department of Neuroradiology, Cannizzaro Hospital, Via Messina, 95126 Catania, Sicily Italy

**Keywords:** Sturge weber syndrome, Isolated leptomeningeal angiomatosis, Magnetic resonance, Perfusion imaging, Digital subtraction angiography

## Abstract

**Background:**

Sturge-Weber syndrome (SWS) is primarily diagnosed in pediatric population, but clinical presentation in late adulthood is rarely reported. Evolution of radiological findings in the adulthood variant of SWS with isolated leptomeningeal angiomatosis has never been reported to our knowledge.

**Case presentation:**

We report here a case of an isolated temporo-parieto-occipital leptomeningeal angiomatosis on the right cerebral hemisphere in a sixty-two-year-old male who presented with generalized seizure, GCS score 14/15 (E4 V4 M6) with equal and reacting pupils, psychomotor slowing, left hemineglect and grade 4 power in the left upper and lower limbs. Over a period of 48 h his neurological status deteriorated, but recovered spontaneously over a week on titration with anticonvulsants. He had a prior history of treatment for focal leptomeningitis, three years ago. Cerebrospinal fluid (CSF) analysis showed glucose of 75 mg/dL, proteins of 65 mg/dL and culture grew no organisms. On follow-up, he had intermittent episodes of focal seizure for two years. Initial, computed tomography of brain showed hyperdense lesion in the parieto-occipital convexity subarachnoid space on the right cerebral hemisphere mimicking subarachnoid hemorrhage and computed tomography angiography showed no significant abnormality. Magnetic resonance imaging (MRI) of brain showed intense pial enhancement in the right temporo-parieto-occipital region with a subtle T2W hyperintense signal in the underlying subcortical white matter without edema or infarct or mass effect. Digital subtraction cerebral angiography (DSA) showed hypertrophy of the cerebral arteries, arteriolo-capillary bed and venules in the right temporo-parieto-occipital territory associated with early arterio-capillary and venous opacification. Serial MRI done after six months, one and two years showed increase in the T2W hyperintense signal in the subcortical white matter and cortical atrophy with no changes in the pial enhancement. MR perfusion imaging showed reduced cerebral blood flow (CBF) and cerebral blood volume (CBV) in the right parieto-temporo-occipital cortical and subcortical regions and increased perfusion in the leptomeninges with reduction of the NAA / Cr ratios in spectroscopy.

**Conclusion:**

Conglomeration of various radiological findings in MRI, Perfusion, MRS and DSA with the clinical presentation can aid in establishing the diagnosis of this rare presentation of SWS-type 3 variant in late adulthood.

## Background

Leptomeningeal angiomatosis, facial port-wine stain in trigeminal distribution and choroidal angioma are the classical features of Sturge-Weber syndrome (SWS) [1]. This is primarily diagnosed in pediatric population and clinical variants like absent facial port-wine stain are also well described [2–9]. Clinical presentation of this syndrome in late adulthood is rarely reported [8]. All the reported cases in adulthood are without facial port-wine stain but with specific typical characteristic neuroradiological findings [8–10]. However, evolution of these radiological findings in the adulthood variant of SWS has never been reported. Here, we report the first case of adulthood variant of SWS in the sixth decade of life and evolution of the radiological findings over two years.

## Case presentation

**Fig. 1:**
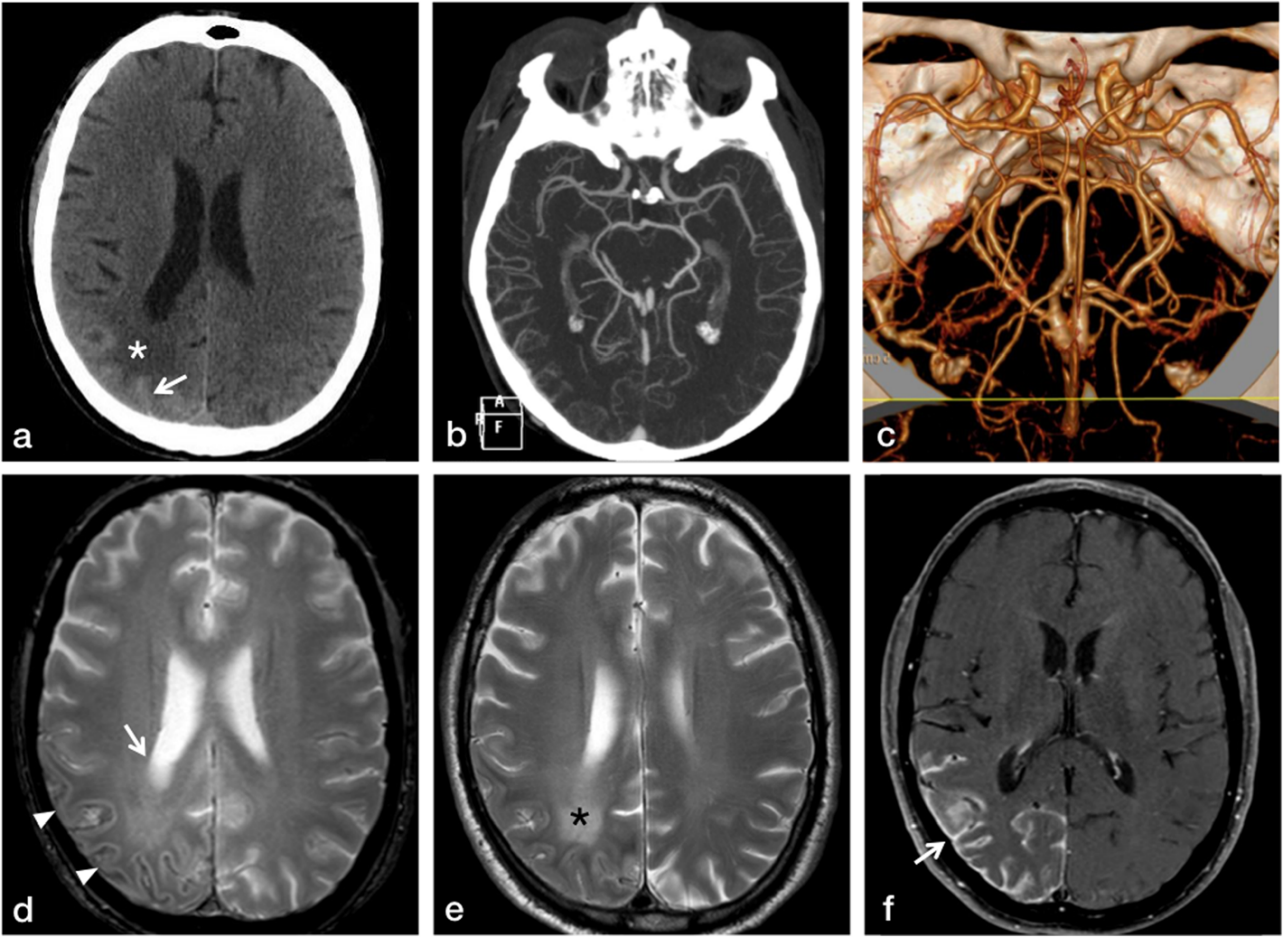
CT (**a**), CTA (**b-c**) and MRI brain (**d-f**). Axial non-enhanced CT (**a**) shows hyperdense lesion in the right parietal convexity subarachnoid spaces (arrow) associated with hypo-attenuation of subcortical and deep white matter (white asterisk). Maximum Intensity Projection (**b**) and Volume Rendering (**c**) from CTA show no significant abnormalities. Axial T2-weighted Gradient Echo (GRE) (**d**) and Turbo Spin Echo (TSE) images (**e**) depict curvilinear hypointensity along the right parietal convexity (arrowheads) with hyperintense signal in underlying deep white matter (black asterisk) and subtle enlargement of lateral ventricle (white arrow). Postgadolinium axial Spin Echo (SE) T1-weighted image (**f**) shows intense serpentine leptomeningeal enhancement along the sulci and gyri of the right parietal lobe (white arrow)

A 62-year-old male was brought to the Emergency unit with one episode of generalized seizures and altered sensorium. He had a prior history of seizure and loss of consciousness three years ago which was treated, elsewhere as focal leptomeningitis of right parieto-occipital region. He had no neurocutaneous markers. His GCS score was 14/15 (E4 V4 M6) with equal and reacting pupils, psychomotor slowing, left hemineglect and grade 4 power in the left upper and lower limbs. His serum electrolytes and blood glucose levels were normal. Computed tomography (CT) of brain (Fig. 1a) showed hyperdense lesions in the parieto-occipital convexity subarachnoid space over the right cerebral hemisphere. CT angiography showed no abnormality (Fig. 1b & 1c).

Over a period of 48 h, his neurological status deteriorated. Magnetic Resonance Imaging (MRI) brain with gadolinium (Fig. 1d-f) at 1.5 T **(**Philips Achieva, Best, The Netherlands) showed intense leptomeningeal enhancement in the right temporo-parieto-occipital region with a subtle T2W hyperintense signal in the underlying subcortical white matter without edema or infarct or mass effect.

Cerebrospinal fluid (CSF) analysis showed glucose of 75 mg/dL, proteins of 65 mg/dL and no abnormal cells were detected ruling out infection, granulomatous inflammation and meningeal carcinomatosis. Electroencephalography (EEG) showed spike and wave epileptic discharges over the right parieto-temporal hemisphere. Anticonvulsants were titrated till suppression of the epileptic discharges in EEG. His sensorium improved over a period of 72 h and he attained a GCS score of 15/15 with left hand grip weakness and impaired rapid alternating movements in the left upper limb. Ophthalmological examination showed no choroid angioma.

**Fig. 2:**
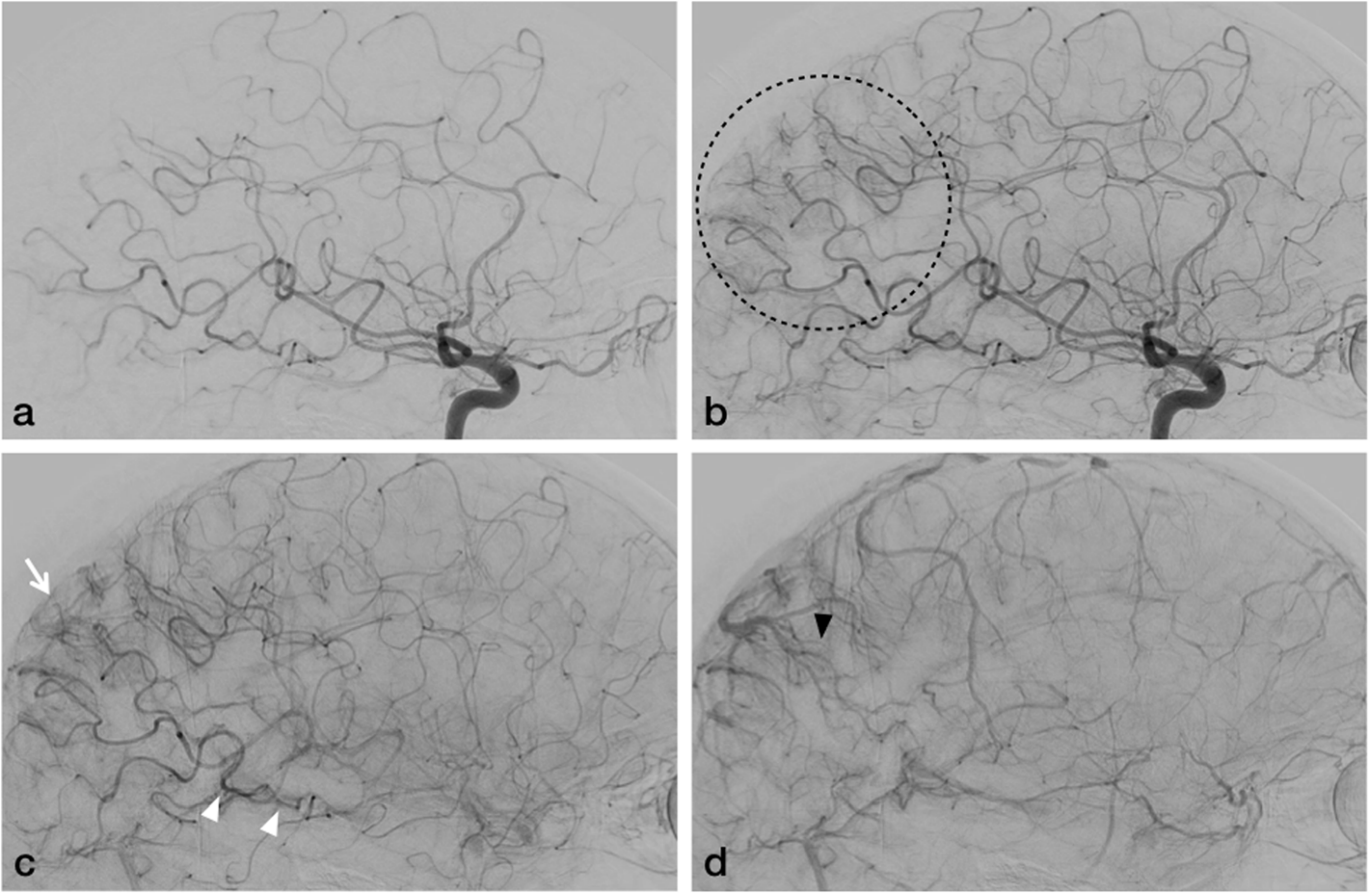
DSA lateral view of right ICA injection during early (**a**) and late arterial phases (**b**), capillary phase (**c**) and early venous phase (**d**). Hypertrophy of distal branches of the cerebral arteries (arrowheads), arteriolo-capillary bed (arrow) in the right temporo-parieto-occipital territory (dashed circle) with early opacification of veins and enlargement of venules (black arrowhead)

Digital subtraction cerebral four vessel angiography with bilateral external carotid injections (Fig. 2) showed hypertrophy of the cerebral arteries, arteriolo-capillary bed and venules in the right temporo-parieto-occipital territory associated with early arterio-capillary and venous opacification, suggesting leptomeningeal angiomatosis. There was no evidence of arteriovenous malformation or fistula.

He was discharged on anticonvulsants with a GCS score of 15/15, reduced attention span, visuospatial impairment, anosognosia, left hemineglect and minimal left-hand grip weakness. He was on regular follow-up for a period of two years, during this period he had intermittent episodes of focal motor seizures involving the left-hand requiring titration of doses of anticonvulsants. His cognitive functions remained status quo and persisted to have left hand grip weakness.

**Fig. 3:**
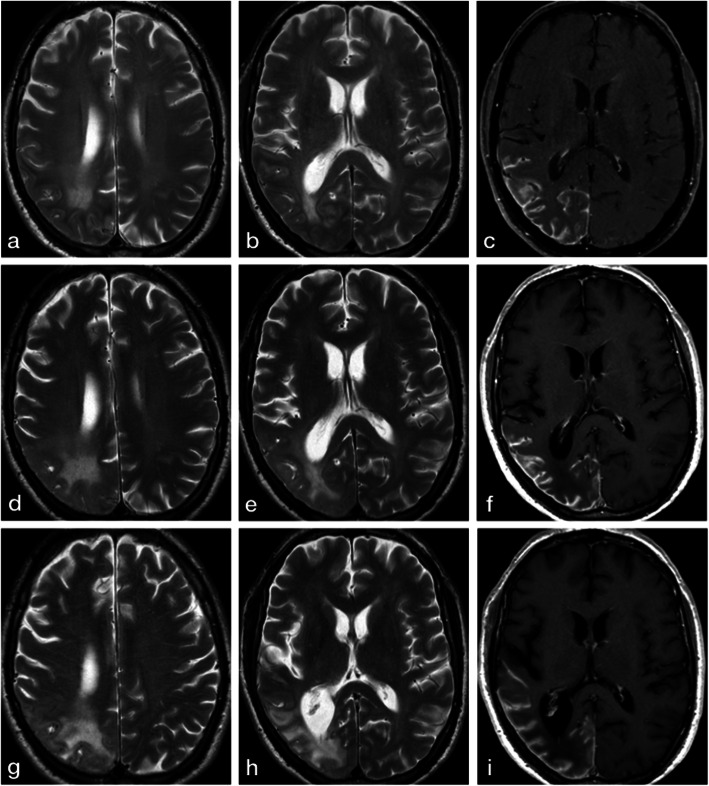
MRI brain demonstrating evolution of neuroradiological findings in the right temporo-parieto-occipital region at six months (**a**-**c**), one-year (**d**-**f**) and two years (**g**-**i**) follow-up. Notice the temporal progression of hyperintense signal changes in the subcortical white matter, subsequent cortical atrophy and ex-vacuo dilation of lateral ventricle in axial T2-weighted Turbo Spin Echo (TSE) images (**a**-**b**, **d**-**e**, **g**-**h**) and stable pial enhancement in axial T1-weighted Spin Echo (SE) images after gadolinium injection (**c**, **f**, **i**)

MRI brain with gadolinium (Fig. 3) at 1.5 T **(**Philips Achieva, Best, The Netherlands), done at six months, one-year and two years after the discharge from the hospital showed increase in the T2W hyperintense signal in the subcortical white matter and cortical atrophy with no changes in the pial enhancement.

**Fig. 4:**
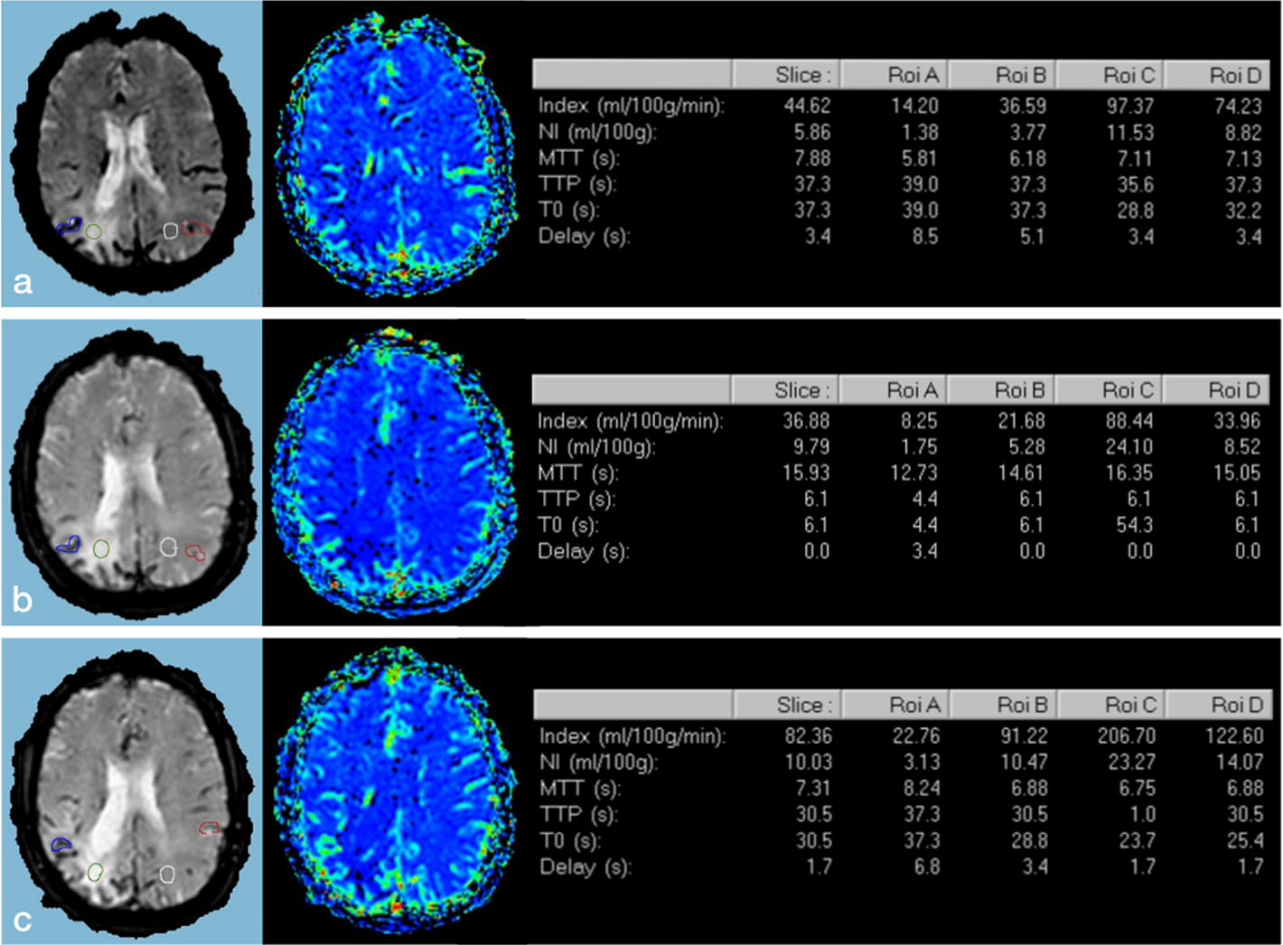
MRI perfusion maps and tables (Extended MR WorkSpace 2.6.3.5, Philips Medical Systems, Best, The Netherlands) at six months **(a)**, one-year **(b)** and two years **(c)** follow-up. Notice reduced cerebral blood flow (CBF) and cerebral blood volume (CBV) in the right parietal cortical and subcortical white matter (green ROI) compared with contralateral normal hemisphere (white ROI) and increased leptomeningeal perfusion on the right side (blue ROI) compared to the left (red ROI)

MR perfusion imaging (T2*echo planar imaging) (Fig. 4) done at six months, one-year and two years follow-up showed reduced cerebral blood flow (CBF) and cerebral blood volume (CBV) in the right parieto-temporo-occipital cortical and subcortical regions compared with contralateral normal hemisphere and increased perfusion in the leptomeninges.

**Fig. 5:**
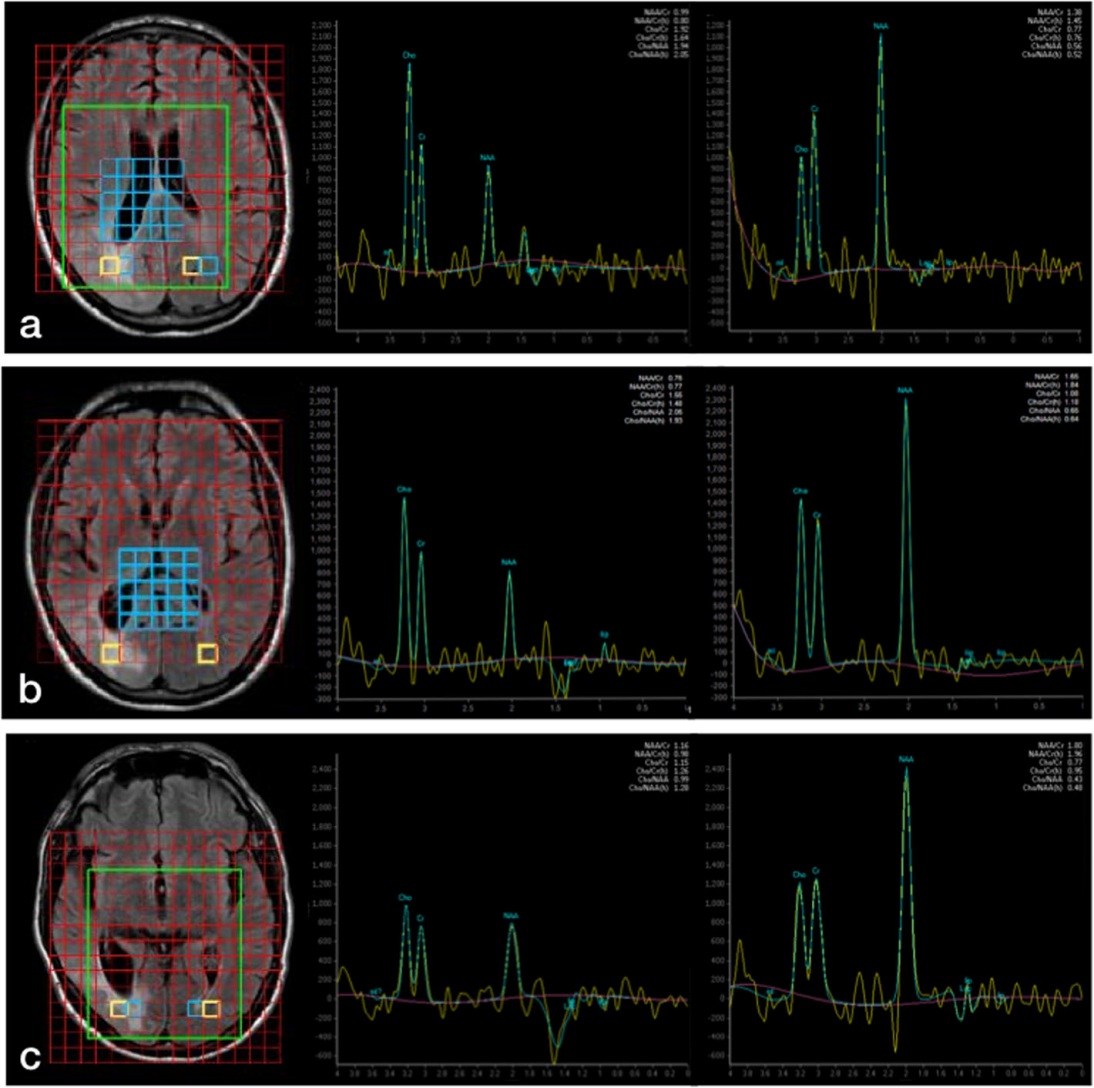
Multi-voxel MR spectroscopy (TE = 144 ms) (Extended MR WorkSpace 2.6.3.5, Philips Medical Systems, Best, The Netherlands) at six months (**a**), one-year (**b**) and two years (**c**) follow-up. Right parietal region shows progressive NAA decline, reduction of NAA/Cr ratio and similar Cho/Cr ratio compared to the left hemisphere

Multi-voxel Magnetic Resonance Spectroscopy (MRS) (TE = 144 ms) (Fig. 5) done at six months, one-year and two years follow-up from the subcortical white matter region with T2 hyperintense signal showed reduction in the NAA/Cr ratio in spectroscopy and preserved Cho/Cr ratio.

## Discussion and conclusion

SWS is a rare sporadic neurocutaneous syndrome with facial port wine stain involving the first division of the trigeminal nerve, ipsilateral leptomeningeal angioma and angioma involving the ipsilateral eye [1]. This is predominantly a syndrome of pediatric population, rarely presents in adulthood. Although the adulthood variant of SWS has been reported by some authors, this would be the first reported case presenting in the sixth decade of life [8–10].

Variants of SWS includes facial and leptomeningeal angioma with or without choroid angioma (Type I), facial nevus and choroid angioma without clinical or imaging signs of cerebral angiomatosis (Type 2), leptomeningeal and choroid angioma without facial nevus or isolated cerebral and leptomeningeal angiomatosis (Type 3) [11, 12]. All the reported cases of adulthood variant of SWS including our case have no facial nevus which could be the possible reason for late presentation and identification of these cases [8–10].

Seizure is the most common neurological manifestation of SWS, affecting around 75 to 90% of all patients [13]. Only 7% of all SWS patients experience seizures after the age of 5 years. Stroke like episodes, paroxysmal paralysis, transient hemiparesis and migraine are other neurological manifestations of SWS [13]. Although the incidence of seizures in SWS is less after five years of age, our case as well as those reported by Zhang et al. and Ishikawa et al., presented with seizures in adulthood. Neurological manifestations in SWS and its variants are due to leptomeningeal angiomatosis leading to chronic hypoxia of the underlying cortical and subcortical brain tissue. Pathological changes like atrophy, neuronal loss, astrogliosis, dysgenic cortex and calcifications in cortical layers can be observed in the affected areas [14]. In our case, this was evident in conventional MRI and testified with the MRS and MR perfusion findings.

Progressive hypoperfusion and glucose hypometabolism leading to neurological deterioration in SWS was demonstrated radiologically by Maria et al. [15] In our case, we noticed hypoperfusion and hypoxic changes in the form of T2W hyperintense signal in the subcortical white matter beneath the region of leptomeningeal angiomatosis as demonstrated by Miao et al. [16] After the initial episode, our patient had multiple episodes of focal motor seizures over two year, requiring titration of anticonvulsants. MRI showed increase in the size of T2W hyperintense signal change in the underlying white matter. This was similar to the observations noted by Miao et al. that chronic seizures are associated with severe white matter changes and low brain perfusion [16]. In our case, MR perfusion and MRS aided in demonstrating the hypoperfusion and hypoxic changes in the brain tissue underlying the leptomeningeal angiomatosis.

Cerebral angiography in SWS demonstrates aberrant pattern of both the arterial and venous cerebral circulation. Arterial thrombosis, abnormal venous drainage, paucity of superficial draining veins, venous occlusions and alternative venous drainage through deep subependymal channels can be observed in SWS [7]. But in our case, we noted hypertrophy of the cerebral arteries, arteriolo-capillary bed, venules, early arterio-capillary and venous opacification in the right temporo-parieto-occipital territory. There was no anomalous venous drainage. Diagnosing isolated leptomeningeal angiomatosis in late adulthood is challenging. Various radiological characteristics with different modalities using MRI, perfusion, MRS and cerebral angiography can aid in establishing the diagnosis of Sturge Weber Syndrome type 3 after excluding infection, granulomatous inflammation and meningeal carcinomatosis. Even in our patient the initial episode of focal leptomeningitis, treated in another center three years ago, could probably be a misinterpretation of the focal leptomeningeal angiomatosis.

This is the first reported case of isolated leptomeningeal angiomatosis presenting in the sixth decade of life with description of radiological findings and its evolution. Conglomeration of various radiological findings in MRI, Perfusion, MRS and DSA with the clinical presentation can aid in establishing the diagnosis of Sturge Weber Syndrome-type 3 variant.

## Data Availability

Not applicable.

## Abbreviations

SWS: Sturge-Weber Syndrome
CT: Computer Tomography
MRI: Magnetic Resonance Imaging
CSF: Cerebrospinal Fluid
EEG: Electroencephalography
CBF: Cerebral Blood Flow
CBV: Cerebral Blood Volume
MRS: Magnetic Resonance Spectroscopy
DSA: Digital Subtraction Angiography

## References

[1] Bendl BJ, Bashir R, Dowling AD (1983). Sturge-Weber syndrome. Cutis.

[2] Taly AB, Nagaraja D, Das S, Shankar SK, Pratibha NG (1987). Sturge-Weber-Dimitri disease without facial nevus. Neurology.

[3] Hatfield M, Muraki A, Wollman R, Hekmatpanah J, Mojtahedi S, Duda EE (1988). Isolated frontal lobe calcification in Sturge-Weber syndrome. AJNR Am J Neuroradiol.

[4] Maiuri F, Gangemi M, Iaconetta G, Maiuri L (1989). Sturge-Weber disease without facial nevus. J Neurosurg Sci.

[5] Aydin A, Cakmakci H, Kovanlikaya A, Dirik E (2000). Sturge-Weber syndrome without facial nevus. Pediatr Neurol.

[6] Jordan PR, Iqbal M, Prasad M (2016). Sturge-Weber syndrome type 3 manifesting as ‘Status migrainosus.’. BMJ Case Rep.

[7] Juhasz C, Chugani HT (2007). An almost missed leptomeningeal angioma in Sturge-Weber syndrome. Neurology.

[8] Terdjman P, Aicardi J, Sainte-Rose C, Brunelle F (1991). Neuroradiological findings in Sturge-Weber syndrome (SWS) and isolated pial angiomatosis. Neuropediatrics.

[9] Pavone P, Praticò AD, Gentile G, Falsaperla R, Iemmolo R, Guarnaccia M (2016). A neurocutaneous phenotype with paired hypo- and hyperpigmented macules, microcephaly and stunted growth as prominent features. Eur J Med Genet.

[10] Ishikawa H, Ii Y, Niwa A, Matsuura K, Maeda M, Tomimoto H (2017). A case of 55-year-old man with first-ever generalized seizure diagnosed with Sturge-Weber syndrome type III by characteristic MRI findings. Rinsho Shinkeigaku.

[11] Gomez-Moreno M, Murrieta-Urruticoechea C, Martinez-Acebes E, Gordo-Manas R (2015). Adult diagnosis of temporo-occipital leptomeningeal angiomatosis. Neurologia (Barcelona, Spain).

[12] Zhang R, Chen W, Hu Q (2014). Shrestha S [Adult Sturge-Weber syndrome without facial hemangioma: report of one case]. Zhejiang Da Xue Xue Bao Yi Xue Ban.

[13] Comi AM, Fischer R, Kossoff EH (2003). Encephalofacial angiomatosis sparing the occipital lobe and without facial nevus: on the spectrum of Sturge-Weber syndrome variants?. J Child Neurol.

[14] Martinez-Bermejo A, Tendero A, Lopez-Martin V, Arcas J, Royo A, Polanco I (2000). Occipital leptomeningeal angiomatosis without facial angioma. Could it be considered a variant of Sturge-Weber syndrome?. Rev Neurol.

[15] Roach ES (1992). Neurocutaneous syndromes. Pediatr Clin North Am.

[16] Pascual-Castroviejo I, Pascual-Pascual S-I, Velazquez-Fragua R, Viano J (2008). Sturge-Weber syndrome: study of 55 patients. Can J Neurol Sci.

[17] Sinawat S, Auvichayapat N, Auvichayapat P, Yospaiboon Y, Sinawat S (2014). 12-year retrospective study of Sturge-Weber syndrome and literature review. J Med Assoc Thai.

[18] Norman MG, Schoene WC (1977). The ultrastructure of Sturge-Weber disease. Acta Neuropathol.

[19] Simonati A, Colamaria V, Bricolo A, Bernardina BD, Rizzuto N (1994). Microgyria associated with Sturge-Weber angiomatosis. Childs Nerv Syst.

[20] Maria BL, Neufeld JA, Rosainz LC, Drane WE, Quisling RG, Ben-David K (1998). Central nervous system structure and function in Sturge-Weber syndrome: evidence of neurologic and radiologic progression. J Child Neurol.

[21] Miao Y, Juhasz C, Wu J, Tarabishy B, Lang Z, Behen ME (2011). Clinical correlates of white matter blood flow perfusion changes in Sturge-Weber syndrome: a dynamic MR perfusion-weighted imaging study. AJNR Am J Neuroradiol.

